# Transforming Growth Factor-α Improves Memory Impairment and Neurogenesis Following Ischemia Reperfusion

**Published:** 2014-10-04

**Authors:** Hassan Alipanahzadeh, Mansooreh Soleimani, Sara Soleimani Asl, Bagher Pourheydar, Ali Nikkhah, Mehdi Mehdizadeh

**Affiliations:** 1 Department of Anatomy, Faculty of Medicine, Uhanto University, Kabol, Afghanestan; 2 Cellular and Molecular Research Center, Faculty of Medicine, Iran University of Medical Sciences, Tehran, Iran; 3 Department of Anatomy, Faculty of Medicine, Iran University of Medical Sciences, Tehran, Iran; 4 Neurophysiology Research Center, Hamadan University of Medical Sciences, Hamadan, Iran; 5 Department of Anatomy, Faculty of Medicine, Hamadan University of Medical Sciences Hamadan, Iran; 6 Neurophysiology Research Center, Department of Anatomy, Faculty of Medicine, Urmia University of Medical Sciences, Urmia, Iran; 7 Cellular and Molecular Research Center, Faculty of Advanced Technologies in Medicine, Department of Anatomy, Iran University of Medical Sciences, Tehran, Iran

**Keywords:** Ischemia-Reperfusion, Hippocampus, Spatial Memory Disorder, TGF-α

## Abstract

**Objective:**

Stroke is most important cause of death and disability in adults. The hippocampal CA1 and sub-ventricular zone neurons are vulnerable to ischemia that can impair memory and learning functions. Although neurogenesis normally occurs in the dentate gyrus (DG) of the hippocampus and sub-ventricular zone (SVZ) following brain damage, this response is unable to compensate for severely damaged areas. This study aims to assess both neurogenesis and the neuroprotective effects of transforming growth factor-alpha (TGF-α) on the hippocampus and SVZ following ischemia-reperfusion.

**Materials and Methods:**

In this experimental study, a total of 48 male Wistar rats were divided into the following groups: surgical (n=12), phosphate buffered saline (PBS) treated vehicle shams (n=12), ischemia (n=12) and treatment (n=12) groups. Ischemia was induced by common carotid occlusion for 30 minutes followed by reperfusion, and TGF-α was then injected into the right lateral ventricle. Spatial memory was assessed using Morris water maze (MWM). Nestin and Bcl-2 family protein expressions were studied by immunohistochemistry (IHC) and Western blot methods, respectively. Finally, data were analyzed using Statistical Package for the Social Sciences (SPSS, SPSS Inc., Chicago, USA) version 16 and one-way analysis of variance (ANOVA).

**Results:**

TGF-α injection significantly increased nestin expression in both the hippocampal DG and SVZ areas. TGF-α treatment caused a significant decrease in Bax expression and an increase in Bcl-2 anti-apoptotic protein expression in the hippocampus. Our results showed a significant increase in the number of pyramidal neurons. Memory also improved significantly following TGF-α treatment.

**Conclusion:**

Our findings proved that TGF-α reduced ischemic injury and played a neuroprotective role in the pathogenesis of ischemic injury.

## Introduction

Stroke, or cerebral ischemia, is one of the most important causes for mortality worldwide (1). Cerebral ischemia reduces blood flow, oxygen and metabolites, with an increase in free radicals. Specific areas of the brain and certain types of neurons such as hippocampal CA1 pyramidal neurons are more sensitive to brain ischemia.

The hippocampus is a key area of the brain cortex involved in memory and learning functions; it plays an important role in the formation of new memory an d in spatial data analysis(2).

During the 1960s, Altman and Das observed the production of new neuronal cells in different areas of the adult brain, including the dentate gyrus (DG) and sub-ventricular zone (SVZ) (3). Later, researchers using Brdu-labeling techniques have found that neurogenesis occurs throughout the developmental period in two areas of the brain, which include the DG and SZV(4). According to studies, following hippocampal damage from disease, traumatic ischemia and injuries, internal antioxidants cause an increase in neurogenesis in the DG and SVZ(5). Increasing neurogenesis in the adult brain, SVZ and DG areas, of many mammalian species may cause morphological and functional improvements after brain ischemia, trauma or primary degenerative injury (6). However, this internal response is unable to compensate for the incurred damage. Therefore, factors affecting the rate of adult neurogenesis in DG and SVZ can be considered as effective strategies to repair hippocampal injur y following ischemia-reperfusion.

Transforming growth factor alpha (TGF-α) is a member of the epidermal growth factor (EGF) family that activates EGF-receptor (EGF-R) trans-membrane tyrosine kinase. These changes include increases in intracellular calcium levels, glycolysis and expression of certain genes, such as the EGFR gene that ultimately lead to DNA synthesis and cell proliferation (7). TGF-α is recognized as the most abundant EGF-R ligand in adult central nervous system (CNS) development, and it is abundant in the striatum, olfactory bulb, hippocampal SVZ and brain stem (8). It has been reported that TGF-α stimulates neuronal differentiation *in vitro* (9), and improves neurogenesis in a Parkinson’s disease model accompanied by behavioral improvement (10-11). In this study, we attempt to determine the effects of TGF-α on neural stem cells of the hippocampal DG and SVZ areas.

## Materials and Methods

A total of 48 adult male Wistar rats were obtained from Pasteur Institute, Iran. The rats were allowed to acclimatize to the colony room for one week prior to conducting the experiments. Rats were maintained in a constant temperature of 21 ± 1˚C (50 ± 10% humidity) on a 12-hour light/12-hour dark cycle with ad libitum access to water and food. All experimental procedures were performed in accordance with the guidelines of the Ethical Committee of Iran University of Medical Sciences.

Animals were randomly assigned to the following four groups:

1. Surgical sham group (n=12) that just underwent surgery with no ischemia and stereotaxic procedures
2. Phosphate buffered saline (PBS) treated vehicle group (n=12) that underwent stereotaxic surgery and received 5 μl PBS in the right lateral ventricle (5 days after surgery) according to Paxinos atlas (12).
3. Ischemia group (n=12) that underwent ischemiareperfusion procedure.
4. Treatment group (n=12) that underwent both the stereotaxic surgery and ischemia procedure and received 50 ng TGF-α in the lateral ventricle (5 days after surgery) according to Paxinos atlas (12).

A study has also showed that nestin expression is maximum 12 days after neurogenesis induction (13) and two months following induction, indicating that the synaptogenesis becomes functional (5). All groups were further subdivided into two groups of 6 rats each; therefore, after 12 days, one of sub-groups was used to assess nestin expression and to perform histological study.

### Surgical procedures

The initial surgical procedure was the same for all animals. We used an intraperitoneal injection of a mixture of ketamine (Rotexmedica, Germany) (100 mg/kg) and xylazine (Sanofi, France) (10 mg/kg) to anesthetize the rats. Rectal temperature was maintained at 37 ± 0.5˚C throughout the experiment. Vertebral arteries were permanently occluded by an electrocuter through the alar foramina of the first cervical vertebra. After a midline cervical incision and muscle dissection, the right and left common carotid arteries (CCA) were exposed, leaving the vagus nerve intact. Transient bilateral ligation of the CCA was performed by clamping for 30 minutes (6).

### Morris water maze (MWM)

Two month after neurogenesis induction, we assessed the spatial memory using the Morris water maze (MWM) test. The MWM was a circular, blackpainted pool (183 cm in diameter and 60 cm in height) which was filled to a depth of 25 cm with water (22± 1℃). The pool was divided into four quadrants with four starting locations, north (N), east (E), south (S) and west (W), and placed at equal distances along the rim.

An invisible platform (10 cm diameter) made of Plexiglass was located 1 cm below the water in the center of the north quadrant. The animals were trained for three days at the same time (10-12 AM), approximately. Each training day consisted of two blocks, with four trials. The time limit per animal was 90 seconds, a time limit of 30 seconds was allowed for the time spent on the platform. The rats rested for 5 minutes between two consecutive blocks.

A video camera linked to a computer was mounted directly above the water maze pool. The time to reach the hidden platform (escape latency), length of the swim path (traveled distances) and percentage of spent time in the target quarter for each rat were recorded. The next day after the last learning trial, we gave each rat a single 60-second probe trial and a visible test. Probe trials were performed without the platform. In the visible tests, the platform used was covered with aluminum foil.

### Histological procedure

For light microscopic study, rats were perfused with 4% paraformaldehyde (Merck, Germany) in 0.1 M phosphate buffer (Merck, Germany) (pH=7.3) and the hippocampi were serially sectioned into 10 μm coronal sections. After deparaffinization and rehydration, sections were stained in 0.1% cresyl violet (Sigma Aldrich, St. Louis, MO, USA) for 3 minutes after which sections were dehydrated, cleared and mounted for light microscopic visualization. The samples were studied under a light microscope (Olympus Provis, Ax70, Japan) and the images were photographed by an digital camera (Olympus, DP 11, Japan). The number of CA1 intact neurons with clearly defined cell bodies and nuclei was counted using a 1000 μm^2^ counting frame. Dark cells or dead neurons were characterized by neuronal shrinkage and cytoplasmic hyperstainability (14). For each animal, the average of neuronal counts was obtained by counting five serial sections at ×400 magnification.

### Immunohistochemistry

Immunohistochemistry (IHC) staining method was performed to visualize nestin as a neural stem cellspecific marker.

Sections were cleared, hydrated and endogenous peroxidase was blocked by the application of hydrogen peroxide (H_2_O_2_). Next, we placed slides in a 10% H_2_O_2_ solution for 10 minutes in the dark. Slides were microwaved for antigen retrieval in citrate buffer (pH=6) for 10 minutes. Unspecific antigens were blocked with bovine serum albumin (BSA), followed by the application of goat polyclonal anti nestin primary antibody (1:250, Sigma Aldrich, MO, USA) for 1 hour. After washing, secondary antibody (1:5000, Sigma Aldrich, MO, USA) was applied for 1 hour. Again slides were washed and stained using reagents from a DAB Substrate Kit (Abcam, England) for 10 minutes. As nestin is a cytoplasmic protein, hematoxylin staining was used as a counter stain for neuclear visualization.

### Western blot

Hippocampi were dissected and immediately frozen in liquid N2. The frozen hippocampi were homogenized with ice-cold lysis buffer that contained radio-immunoprecipitation assay (RIPA) buffer and protease inhibitor cocktail (Sigma Aldrich, St. Louis, MO, USA) , at a ratio of 1:10, for 1 hour, then centrifuged at 12000 g for 20 minutes at 4˚C. The supernatant was removed and stored until further analysis. After determining the protein concentration with a Bio-Rad assay system (Bio-Rad, San Francisco, CA, USA), 100 μg aliquots of protein from each sample were denatured with sample buffer that consisted of 6.205 mM Tris-HCl, 10% glycerol, 2% sodium dodecyl sulphate (SDS), 0.01% bromophenol blue and 50 mM 2-mercaptoethanol (2-ME) (Sigma Aldrich, St. Louis, MO, USA) at 95˚C for 5 minutes and then separated using a 10% sodium dodecyl sulfate-polyacrylamide gel electrophoresis (SDS-PAGE) for 90 minutes at 120 voltage. Resultant proteins were transferred to a Hybond-P™ membrane (Amersham Pharmacia Biotech, Piscataway, NJ, USA). Membranes were blocked with 5% nonfat milk dissolved in Tween Tris buffered saline (TTBS) buffer that contained 50 Mm Tris, 1.5% NaCl, and 0.05% Tween 20 at pH=7.5 for 1 hour. Proteins were subsequently stained with anti-Bcl-2 and anti-Bax monoclonal antibodies (1:1000; Sigma Aldrich, MO, USA) for 2 hours followed by staining with alkaline phosphatase-conjugated secondary antibodies (1:10000, Sigma Aldrich, MO, USA) for 1 hour. Bands were detected using a chromogenic substrate, 5-bromo-4-chloro-3-indolyl phosphate in the presence of nitroblue tetrazolium (Sigma Aldrich, St. Louis, MO, USA). Beta-actin antibody (1:1000, Sigma Aldrich, MO, USA) was used as standard for normalization. The bands from various groups that corresponded to the appropriate molecular weight for each subunit were analyzed and values were compared using densitometric measurements by UVIdoc image analysis system (UVIdoc, Houston, TX, USA).

### Statistical analysis

We presented the data as mean ± SEM. Results were analyzed using Statistical Package for the Social Sciences (SPSS, SPSS Inc., Chicago, USA) version 16 and one-way analysis of variance (ANOVA). Post-hoc comparisons were performed using the Tukey test. Value of p≤0.05 was considered statistically significant.

## Results

Since there was no difference between vehicle and surgical sham groups, in the present study, we showed only surgical sham group’s data.

### Protective effect of TGF-α on neuronal density in the CA1 on days 12 and 72 post-surgery

**Fig 1:**
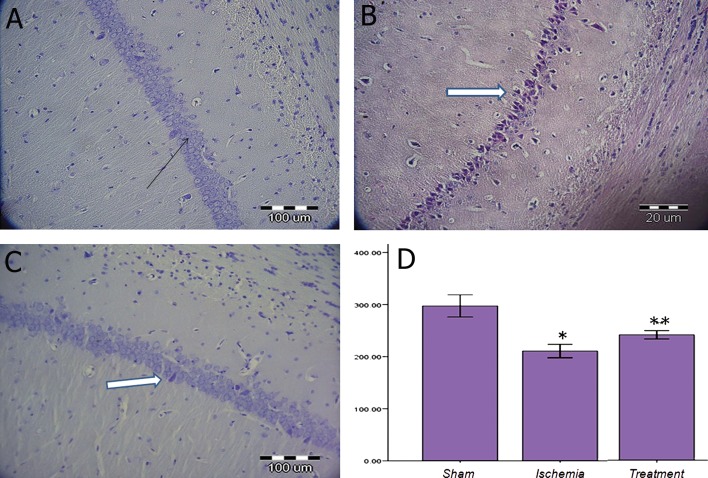
Nissl staining in the sham (A), ischemia (underwent ischemia procedure) (B) and treatment (received TGF-α) (C) groups during 12 days post-surgery. Black arrow shows an intact neuron and white arrow shows the presence of dead cells or dark neuron. Data were presented as mean ± SEM.*; P<0.05 vs. sham group and **; P<0.05 vs. sham and ischemia groups.

**Fig 2:**
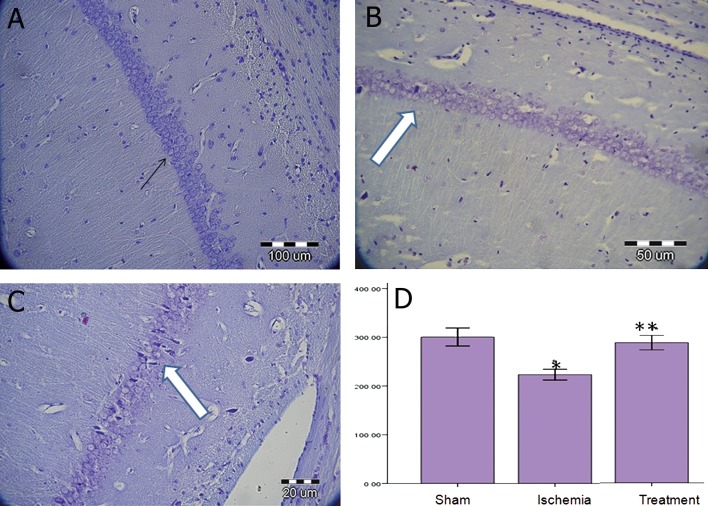
Nissl staining in the sham (A), ischemia (underwent ischemia procedure) (B) and treatment (received TGF-á) (C), groups during 72 days post-surgery. Black arrow shows an intact neuron and white arrow shows the presence of dead cells or dark neurons. Data were presented as mean ± SEM.*; P<0.05 vs. sham group and **; P<0.05 vs. sham and ischemia groups (D).

Our results showed that ischemia-reperfusion significantly decreased neuronal density in the CA1 hippocampus compared to the surgical sham group (p<0.05, Fig 1). TGF-α administration increased neuronal density in the treatment group compared to the ischemia group (p<0.05) on day 12. There was a significant difference in the average neuronal density among the surgical sham, ischemia and treatment groups (p<0.05, Fig 2) during 72 days after surgery.

### Effect of TGF-α on neurogenesis in DG and SVZ areas on day 12 post-surgery

**Fig 3:**
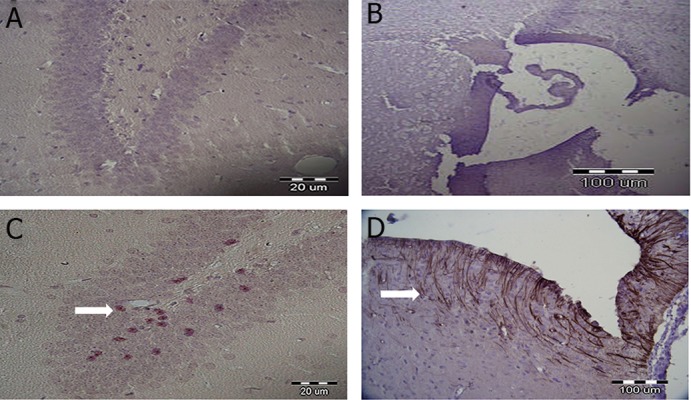
Nestin expression in DG (A and B) and SVZ (C and D) areas using IHC staining method. A and C were used as ischemia groups. White arrows indicate nestin expression. Hematoxylin staining was used as the counter stain (Magnification: ×400).

We used IHC staining method to study expressions of nestin in the DG and SVZ areas of the ischemia and treatment groups. The results indicated that the treatment group had more nestin expression compared with the ischemia group in both the DG (Fig 3A-B) and SVZ (Fig 3C-D) areas.

### Protective effect of TGF-α on ischemia-induced learning impairment in MWM

**Fig 4:**
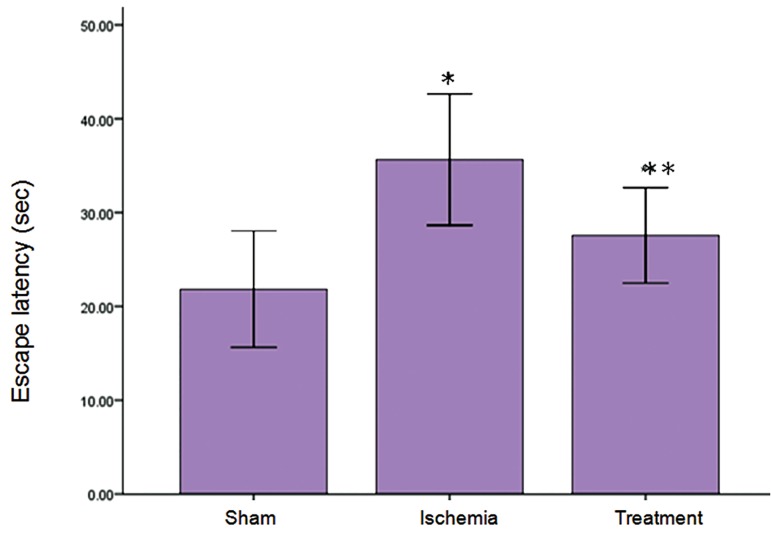
Effect of TGF-α on ischemia-reperfusion-induced escape latency impairment in MWM. Escape latency was analyzed by MWM on the next day following the last administration of TGF-α. Data are presented as mean ± SEM. *; P<0.05 vs. the sham group and **; P<0.001 vs. sham and ischemia groups.

**Fig 5:**
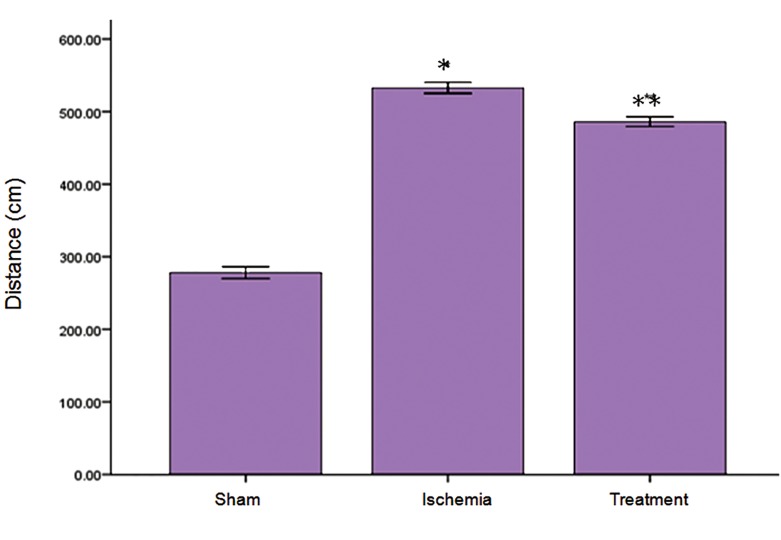
Effect of TGF-α on ischemia-reperfusion-induced increase in distance traveled during the training days according to MWM. Data were presented as mean ± SEM. *; P<0.05 vs. ischemia group and **; P<0.05 vs. ischemia and sham groups.

The results indicated that the mean escape latency significantly increased in ischemia group compared with sham group, while TGF-α treatment caused a reduction escape latency compared to the ischemia group in MWM (p<0.05, Fig 4). There was a significant difference between sham and ischemia groups in traveled distance (p<0.05), while treatment group demonstrated a significant decrease in traveled distance to the escape platform compared to the ischemia group (p<0.05, Fig 5). There were no significant differences in escape latency and traveled distance in the probe trial and visible sessions in the ischemia and treatment groups compared to the sham group (data not shown).

### Protective effect of TGF-α on expression levels of Bax and Bcl-2 proteins

**Fig 6:**
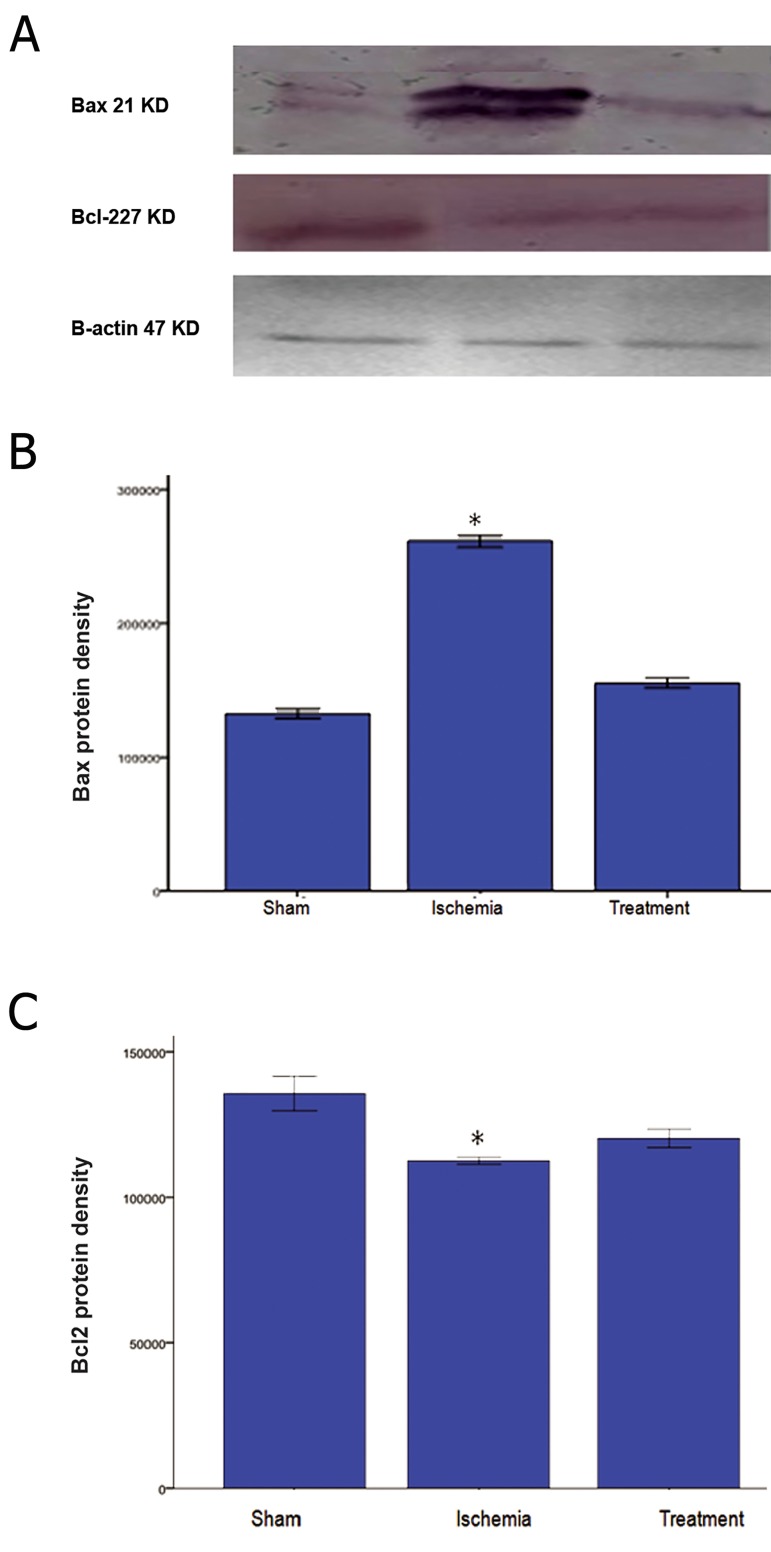
Expression levels of BAX and Bcl-2 proteins in sham, ischemia and treatment groups. Bands were detected by Western blot analysis. TGF-α treatment significantly leads to down- and up- regulation of Bax and Bcl-2, respectively (*; P<0.05 vs. sham group).

Ischemia-reperfusion caused a significant upregulation (p<0.05) of Bax protein expression and a significant down-regulation (p<0.01) of Bcl-2 proteins expression (Fig 6). Although treatment with TGF-α decreased Bax expression in the treatment group compared to the ischemia group, the value was not significant. TGF-α treatment caused insignificant expression of Bcl-2 protein when compared with the ischemia group.

## Discussion

The present study demonstrated memory impairment, apoptosis, and reduction in neurogenesis following ischemia- reperfusion injury. The finding was that the TGF-α administration improved ischemia- induced neurotoxicity.

Scientists have reported that neurogenesis occurs in certain areas of the brain, such as the DG and SVZ, throughout the mammalian lifetime which improve behavioral functions (3). Several line of evidence suggests that a global cerebral ischemia model causes neurodegenration in the hippocampus CA1, neucortex and striarum. Furthermore, it impairs memory and learning ability in rats (15), confirming our findings that ischemia- reperfusion leads to memory impairment in MWM, which is especially sensitive to lesions of the hippocampus (16).

In addition, our results showed that ischemiareperfusion caused an up- regulation of proapoptotic Bax and a down- regulation of anti apoptotic Bcl-2 proteins in the hippocampus, confirming another study that reported cell death following pathological condition such as seizure, toxins, and hyperglycemia (17-19).

In the hippocampus, new CA1 granule cells are formed from precursor cells in the DG and SVZ areas that migrate and differentiate into neural cells of the CA1 (20).

According to Cooper et al., in a rat model of Parkinson’s disease, neural stem cells in the SVZ exposed to TGF-α underwent massive proliferation, migration and differentiation (13). TGF-α blinding to the EGF receptor (EGFR), a tyrosine kinase receptor encoded by ErbB gene, is expressed in the proliferating cells of the developing rat (21). EGFR activates ERKs, leading to the phosphorylation of SMAD1 in the cells. It has been reported that there are synergism between EGF and Sonic Hedgehog (SHH) signaling (22). SHH involves in cell proliferation, growth of postnatal dorsal brain and of adult SVZ and DG areas, as well as enhancement of neurogenesis (23, 24). Unlike the SHH, the BMP signaling inhibits neural differentiation and promotes gliosis (23). Palma et al. reported that synergism between EGF and SHH signaling can promote cell differentiation and neurogenesis, indirectly, through the inhibition of BMP signaling (22). Consistent with other studies, in a rat stroke model, we found intracranial administration of TGF-α induced neural stem cells in the DG and SVZ areas (10). Leker et al. have reported that TGF-α can induce angiogenesis, neurogenesis and neuroprotection following a stroke (25). Guerra-Crespo et al. have also reported that neural stem cells respond to ischemic damage, while behavioral recovery increases considerably after TGF-α administration. This treatment presents as an approach for chronic stroke and other neurological damages in humans (10). The nestin as a cytoplasmic in - termediate filament marker is used to identify neural progenitor and stem cells in the mammalian brain (26), and in this study, we indicated that TGF-α treatment increased the nestin-positive population in ischemic rats that it may directly involve in cellular proliferation.

Consistent with our results, Cooper et al. showed that the TGF-α delivery to a model of Parkinson’s disease induced proliferation and migration of endogenous adult neural progenitor cells (13). Our results suggest that TGF-α may trigger the proliferation of neuronal progenitors, moreover, given the behavioral improvement, these progenitor cells are likely to participate in the generation of new neurons. Guerra-Crespo showed that expression of nestin following intranasal administration of TGF-α in chronic stroke model was less dramatic than the results obtained when it was induced intracranially (27), suggesting that it is susceptible to degradation with a possible short half-life in the nasal environment.

## Conclusion

The current study, in agreement with other studies, showed that ventricular injection of TGF-α (50 ng) in conjunction with stereotaxic surgery significantly increased the number of area CA1 pyramidal cells in the experimental groups compared with the vehicle group on days 12 and 72. There was a significant increase in spatial memory according to the MWM behavior test compared to the day 72 of vehicle group. In addition, there was evidence of increased expression of nestin in the DG and SVZ areas of treatment group on day 12 compared with the vehicle group. We observed reduced expression of Bax (apoptosis protein) along with increased expression of Bcl-2 (anti-apoptotic protein) in the treatment groups compared to the ischemic group.

## References

[1] Camarata PJ, Heros RC, Latchaw RE (1994). Brain attack: the rationale for treating stroke as a medical emergency. Neurosurgery.

[2] Eichenbaum H (2004). Hippocampus: cognitive processes and neural representations that underlie declarative memory. Neuron.

[3] Altman J, Das GD (1965). Autoradiographic and histological evidence of postnatal hippocampal neurogenesis in rats. J Comp Neurol.

[4] Gage FH (2000). Mammalian neural stem cells. Science.

[5] Salazar-Colocho P, Lanciego JL, Del Rio J, Frechilla D (2008). Ischemia induces cell proliferation and neurogenesis in the gerbil hippocampus in response to neuronal death. Neurosci Res.

[6] Schmidt W, Reymann KG (2002). Proliferating cells differentiate into neurons in the hippocampal CA1 region of gerbils after global cerebral ischemia. Neurosci Lett.

[7] Xian CJ, Zhou XF (1999). Roles of transforming growth factor-alpha and related molecules in the nervous system. Mol Neurobiol.

[8] Lazar LM, Blum M (1992). Regional distribution and developmental expression of epidermal growth factor and transforming growth factor-alpha mRNA in mouse brain by a quantitative nuclease protection assay. J Neurosci.

[9] Ezeonu I, Wang M, Kumar R, Dutt K (2003). Density-dependent differentiation in nontransformed human retinal progenitor cells in response to basic fibroblast growth factor- and transforming growth factoralpha. DNA Cell Biol.

[10] Guerra-Crespo M, Gleason D, Sistos A, Toosky T, Solaroglu I, Zhang JH (2009). Transforming growth factor-alpha induces neurogenesis and behavioral improvement in a chronic stroke model. Neuroscience.

[11] Gleason D, Fallon JH, Guerra M, Liu JC, Bryant PJ (2008). Ependymal stem cells divide asymmetrically and transfer progeny into the subventricular zone when activated by injury. Neuroscience.

[12] Paxinos G, Watson C, Pennisi M, Topple A (1985). Bregma, lambda and the interaural midpoint in stereotaxic surgery with rats of different sex, strain and weight. J Neurosci Methods.

[13] Cooper O, Isacson O (2004). Intrastriatal transforming growth factor a delivery to a model of Parkinson’s disease induces proliferation and migration of endogenous adult neural progenitor cells without differentiation into dopaminergic neurons. J Neurosci.

[14] Soleimani Asl S, Mousavizedeh K, Pourheydar B, Soleimani M, Rahbar E, Mehdizadeh M (2013). Protective effects of N-acetylcysteine on 3, 4-methylenedioxymethamphetamine- induced neurotoxicity in male Sprague-Dawley rats. Metab Brain Dis.

[15] McBean DE, Kelly PA (1998). Rodent models of global cerebral ischemia: a comparison of two-vessel occlusion and four-vessel occlusion. Gen Pharmacol.

[16] Soleimani Asl S, Farhadi MH, Naghdi N, Choopani S, Samzadeh-Kermani A, Mehdizadeh M (2011). Nonacute effects of different doses of 3, 4-methylenedioxymethamphetamine on spatial memory in the Morris water maze in Sprague-Dawley male rats. Neural Regen Res.

[17] Nazem A, Jafarian AH, Sadraie SH, Gorji A, Kheradmand H, Radmard M (2012). Neuronal injury and cytogenesis after simple febrile seizures in the hippocampal dentate gyrus of juvenile rat. Childs Nerv Syst.

[18] Ahmadpour SH, Haghir H (2011). Diabetes mellitus type 1 induces dark neuron formation in the dentate gyrus: a study by Gallyas’ method and transmission electron microscopy. Rom J Morphol Embryol.

[19] Soleimani Asl S, Falahati P, Shekar riz N, Molavi N, Esmaeili F, Azimi Z (2011). Protective effects of N-Acetyl-L-cystein on 3, 4-Methylene dioxymethamphetamie- Induced neurotoxicity in cerebellum of male rats. Basic Clin Neurosci.

[20] Hastings NB, Gould E (1999). Rapid extension of axons into the CA3 region by adult-generated granule cells. J Comp Neurol.

[21] Kornblum HI, Hussain RJ, Bronstein JM, Gall CM, Lee DC, Seroogy KB (1997). Prenatal ontogeny of the epidermal growth factor re-ceptor and its ligand, transforming growth factor alpha, in the rat brain. J Comp Neurol.

[22] Palma V, Ruiz i Altaba A (2004). Hedgehog-GLI signaling regulates the behavior of cells with stem cell properties in the developing neocortex. Development.

[23] Lai K, Kaspar BK, Gage FH, Schaffer DV (2003). Sonic hedgehog regulates adult neural progenitor proliferation in vitro and in vivo. Nat Neurosci.

[24] Dahmane N, Sanchez P, Gitton Y, Palma V, Sun T, Beyna M (2001). The Sonic Hedgehog-Gli pathway regulates dorsal brain growth and tumorigenesis. Development.

[25] Leker RR, Toth ZE, Shahar T, Cassiani-Ingoni R, Szalayova I, Key S (2009). Transforming growth factor α induces angiogenesis and neurogenesis following stroke. Neuroscience.

[26] Jiang Y, Henderson D, Blackstad M, Chen A, Miller RF, Verfaillie CM (2003). Neuroectodermal differentiation from mouse multipotent adult progenitor cells. Proc Natl Acad Sci USA.

[27] Guerra-Crespo M, Sistos A, Gleason D, Fallon JH (2010). Intranasal administration of PEGylated transforming growth factor-α improves behavioral deficits in a chronic stroke model. J Stroke Cerebrovasc Dis.

